# LocExpress: a web server for efficiently estimating expression of novel transcripts

**DOI:** 10.1186/s12864-016-3329-3

**Published:** 2016-12-22

**Authors:** Mei Hou, Feng Tian, Shuai Jiang, Lei Kong, Dechang Yang, Ge Gao

**Affiliations:** 1 State Key Laboratory of Protein and Plant Gene Research, College of Life Sciences, Center for Bioinformatics, Peking University, Beijing, 100871 People’s Republic of China; 2 Peking-Tsinghua Center for Life Sciences, Academy for Advanced Interdisciplinary Studies, Peking University, Beijing, 100871 People’s Republic of China

**Keywords:** Expression estimation, Transcriptome, RNA-Seq, Web server

## Abstract

**Background:**

The temporal and spatial-specific expression pattern of a transcript in multiple tissues and cell types can indicate key clues about its function. While several gene atlas available online as pre-computed databases for known gene models, it’s still challenging to get expression profile for previously uncharacterized (i.e. novel) transcripts efficiently.

**Results:**

Here we developed LocExpress, a web server for efficiently estimating expression of novel transcripts across multiple tissues and cell types in human (20 normal tissues/cells types and 14 cell lines) as well as in mouse (24 normal tissues/cell types and nine cell lines). As a wrapper to RNA-Seq quantification algorithm, LocExpress efficiently reduces the time cost by making abundance estimation calls increasingly within the minimum spanning bundle region of input transcripts. For a given novel gene model, such local context-oriented strategy allows LocExpress to estimate its FPKMs in hundreds of samples within minutes on a standard Linux box, making an online web server possible.

**Conclusions:**

To the best of our knowledge, LocExpress is the only web server to provide nearly real-time expression estimation for novel transcripts in common tissues and cell types. The server is publicly available at http://loc-express.cbi.pku.edu.cn.

**Electronic supplementary material:**

The online version of this article (doi:10.1186/s12864-016-3329-3) contains supplementary material, which is available to authorized users.

## Background

The rapid growth of high-throughput RNA-Seq data enables thousands of novel transcripts discovered annually, with the long noncoding RNAs (lncRNAs) as the major repertoire [1]. RNA expression profile provides important functional hints, which is particularly helpful for novel lncRNAs due to their largely elusive mechanisms [2]. While several expression atlas databases [3–5] work well for known gene models, they cannot handle novel transcripts which were not pre-calculated (also see Additional file 1: Table S1). On the other hand, the *ab initio* analysis of raw RNA-Seq data [6–8], which can survey the transcriptome global picture with both known and novel gene models, is time consuming and hardly practical for bench biologists.

Here we propose LocExpress, a local context-oriented expression abundance estimation tool for novel transcripts. For a given gene model, LocExpress estimates its abundance only based on its minimum spanning bundle (MSB) region instead of reanalyzing the whole transcriptome. Such context-oriented strategy enables the nearly real-time expression profiling for a novel transcript in hundreds of samples, with the same accuracy of standard pipelines. To help bench biologists, we made LocExpress publicly available as a web server at http://loc-express.cbi.pku.edu.cn. Currently, the website supports instant abundance estimation across 101 human and mouse samples (Table 1 and 2, also see Additional file 2 for the full sample list).

**Table 1:** LocExpress supports expression estimation in common tissues/cells

	Human	Mouse
Circulatory system	Heart, Whole blood	Heart
Digestive system	Colon, Liver, Pancreas, Stomach	Colon, Duodenum, Large intestine, Liver, Pancreas, Sigmoid, Small Intestine, Stomach
Endocrine system	Subcutaneous adipose, Thyroid	Adipose, Adrenal
Exocrine system	Skin, Breast mammary tissue	
Immune system	Spleen	B cell (CD19+), B cell (CD43-), MEP, Spleen
Nervous system	Cortex, Hippocampus, Substantia nigra	Cerebellum, Cortex
Renal system	Kidney	Bladder, Kidney
Reproductive system	Ovary, Prostate, Testis	Ovary, Testis, Placenta
Respiratory System	Lung	Lung
Skeletal system	Skeletal muscle

**Table 2:** LocExpress supports expression estimation in common cell lines

	Human	Mouse
Normal cell line	B cell (CD20+), GM12878, H1-hESC, HEK293, HMEC, HUVEC, IMR90, CD14+ monocytes	416B, C2C12, CH12, ES-E14, NIH-3 T3, Patski
Cancer cell line	A549, HeLa-S3, HepG2, K562, MCF-7, SK-N-SH_RA	10 T1/2, 416B, MEL

## Implementation

**Fig. 1:**
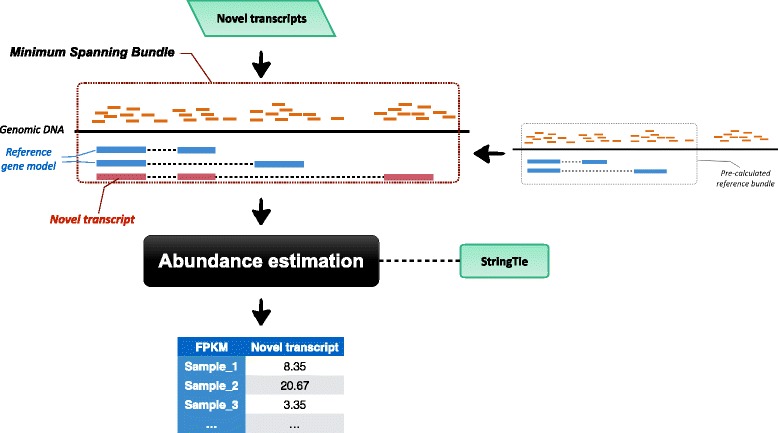
The workflow of LocExpress. For an input novel transcript, LocExpress infers MSB for abundance estimation, and obtain FPKM accordingly

As a wrapper for canonical RNA-Seq quantification algorithms, LocExpress takes full advantage of the locality of RNA-Seq data, and makes the abundance calls increasingly (Fig. 1, also refer to Additional file 3: Figure S2 for detailed workflow ).

The basic idea of LocExpress is straightforward: for a novel transcript, LocExpress will first infer its minimum spanning bundle (MSB), and make the (initial) expression call based on reads within the MSB only. Here, a bundle is defined as a continuous genomic region which is covered by overlapped reads (with no more than 50 bp gaps) as well as known gene models. The MSB is an independent unit in making an expression call (i.e. the expression estimation is independent in each bundle). And a novel transcript’s MSB can be inferred by merging the transcript’s spanning region with (overlapped) reference bundles which was derived by running StringTie [9] on the reference gene model (GENCODE v24 for human and vM9 for mouse, also see Additional file 1 for detailed RNA-Seq analysis procedure). After that, reads and existing gene models within the MSB are extracted and fed into the StringTie code. The resulting relative FPKM in each sample is further corrected for the fragment length by

$$ Corrected\; FPKM=\frac{Relative\; FPKM\times Local\; total\; fragment\; length}{Global\; total\; fragment\; length} $$

Finally, these corrected FPKMs are geometrically normalized across samples, and reported to users, with replicates for the same cell type are averaged before.

All core modules are implemented in Python and Linux Shell, and the LocExpress website is developed based on Ruby on Rails (v4.2.6) with MySQL database supported. LocExpress is publicly available at http://loc-express.cbi.pku.edu.cn.

## Results and discussion

### Performance evaluation

To assess the performance of LocExpress, we simulated multiple user submissions by randomly choosing 300 transcripts from GENCODE reference gene models as “novel” transcripts per sample each time, resulting in 11,317 human transcripts and 9112 mouse transcripts in total. The evaluation was conducted on 40 human samples and 33 mouse samples independently. In each run, the chosen “novel” transcripts were removed from the original reference gene models and feed into the LocExpress one by one. Meanwhile, the output of StringTie ran in quantification-only mode (specified by “-e -B”) with the full GENCODE gene models as reference annotation (specified by “-G”) was taken as the “gold standard” for validating the correctness of LocExpress.

**Fig. 2:**
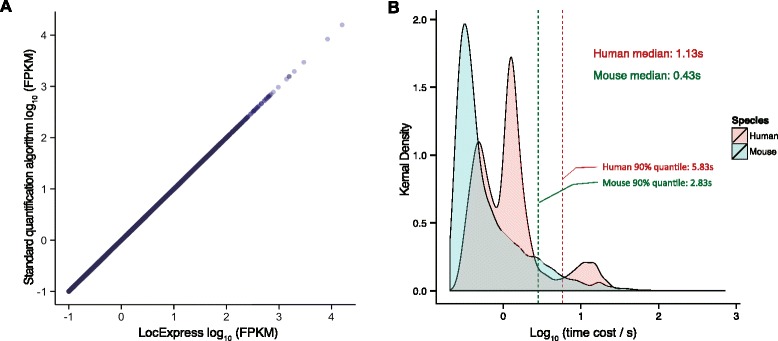
Evaluation of LocExpress. **a** LocExpress arrives the same accuracy as the standard quantification algorithm. **b** The time cost per sample for a novel transcript. The median time cost is only 1.13 s in human and 0.43 s in mouse. The Linux box is configured with two Intel Xeon Processor E5-2670 v2 10C 2.5GHz CPUs, with 4*16GB ECC DDR3 1866 MHz memory

The evaluation shows that the LocExpress can correctly estimate abundance (Fig. 2a) within only seconds: the median time for a novel transcript per sample is 1.13 s in human and 0.43 s in mouse (Fig. 2b). While there are also dozen of extreme cases (9 in human and 17 in mouse) taking more than one minute per sample due to their complicated splicing structures as well as dense reads distribution in their MSB regions, the majority of transcripts takes several seconds only (90% quantile of time cost per sample is 5.83 s in human and 2.83 s in mouse).

To further verify the performance of the LocExpress, we re-ran the evaluation on 3946 newly added transcripts in human GENCODE (v24 vs. v21), and 3874 in mouse GENCODE (vM9 vs. vM7, also see the “Evaluation on newly added transcripts” section in Additional file 1 for more details). Consistently, LocExpress is able to estimate expression abundance correctly for these novel transcripts (Additional file 1: Figure S1A) in nearly real-time (median time 0.71 s for human and 0.53 s for mouse, Additional file 1: Figure S1B).

### User interface

**Fig. 3:**
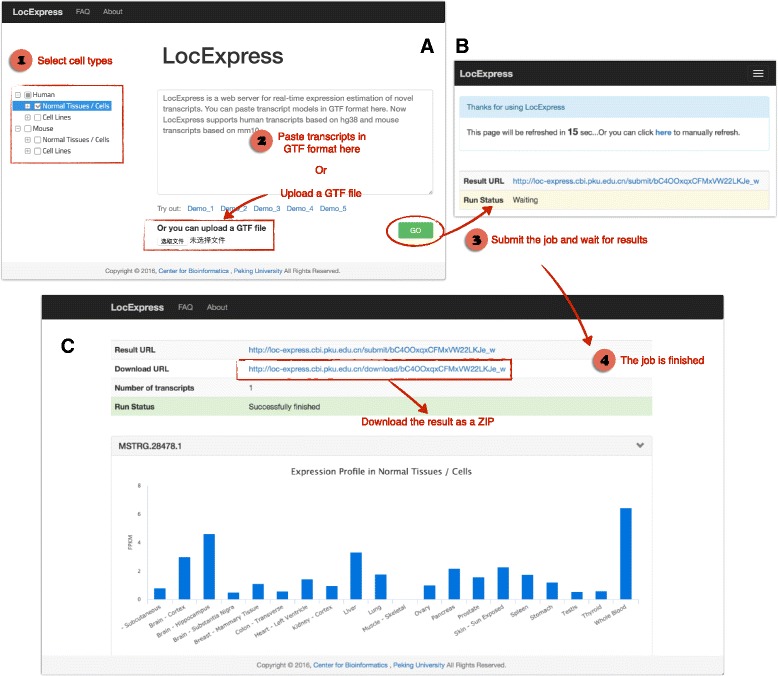
The web interface of LocExpress. LocExpress web server is very convenient to use. **a** The “Home” page. Users can select cell types and submit transcript GTF in this page. **b** The “Run status” page which shows the running status of current submission. **c** The “Result” page. Expression profiles of submitted transcripts are rendered as bar charts

LocExpress is designed to be intuitive. The most common operations (such as submitting transcript GTF and checking results) can be performed with just a few clicks (Fig. 3). Users can select cell types and submit novel transcripts in GTF format at the “Home” page of LocExpress website (Fig. 3a). Then, users are led to the result page directly. Run status is showed on this page. If the job is not finished, this page will self-refresh every 30 s (Fig. 3b). Users can keep the page open and wait the job to be finished, or just record the result URL and come back later. After the job is successfully finished, expression profiles of each transcript are rendered as bar plots (Fig. 3c). Users can also download the calculation results in text format as a ZIP (Fig. 3c).

## Conclusions

To the best of our knowledge, LocExpress is the only web server that can provide efficient real-time expression estimation for novel transcripts across multiple common human and mouse tissues and cell types. Taking advantage of RNA-Seq locality, LocExpress wraps canonical RNA-Seq quantification algorithm, archiving the same accuracy with overwhelming efficiency for novel transcripts. The median time cost is only about one second per submit per sample in human and half second in mouse. Powered by the intuitive web interface, LocExpress could be a useful tool for bench biologists to get the complete expression profile of their interested novel transcripts in just minutes with only a few clicks. In the future, we will continuously improve the LocExpress with more samples and more friendly interface based on users’ feedback.

## Availability and requirements

Project name: LocExpress

Project home page: http://loc-express.cbi.pku.edu.cn

Operating system: LocExpress can be accessed from any platform by using modern Web browsers (recommended but not limited to the latest version of Safari, Chrome and Firefox).

Programming languages: Python, Shell and Ruby

Any restrictions to use by non-academics: For non-academic use, please contact loc-express@mail.cbi.pku.edu.cn.

## Additional files

Additional file 1: This file includes the supplementary table and figures, as well as additional methods and discussion. (DOCX 130 kb)
Additional file 2: This file lists human and mouse datasets used to build the LocExpress web server, including reads mapping information. (XLSX 18 kb)
Additional file 3: This file is Figure S2, a flowchart of detailed workflow of LocExpress. (PDF 332 kb)

## Abbreviations

FPKM: Fragments per kilobase of exon per million fragments mapped
MSB: Minimum spanning bundle
