# Supplementary material for: LocExpress: a web server for efficiently estimating expression of novel transcripts
Source: BMC Genomics. 2016 Dec 22;17(Suppl 13):1023. doi: 10.1186/s12864-016-3329-3 (PMC5260097; doi:10.1186/s12864-016-3329-3)
Supplement: Additional file 1: — This file includes the supplementary table and figures, as well as additional methods and discussion. (DOCX 130 kb) [file 12864_2016_3329_MOESM1_ESM.docx]

# Additional file 1

This file includes the supplementary table and figures, as well as additional methods and discussion.

# Supplementary table

## Table S1. The human reference annotation used in each database / tool for gene expression in normal tissues / cells

| **Databases / Tools** | **Reference annotation** |
| --- | --- |
| Expression Atlas | Ensembl 84 |
| BioGPS  (Barcode on normal tissues dataset ) | U133plus2 Affymetrix |
| GeneCards  (GTEx dataset) | GENCODE v19 |
| LocExpress | Based on GENCODE v24 (Ensembl 84);  Real-time calculation for novel transcripts |

# Supplementary figures

## Figure S1 – Evaluation of LocExpress on newly added transcripts


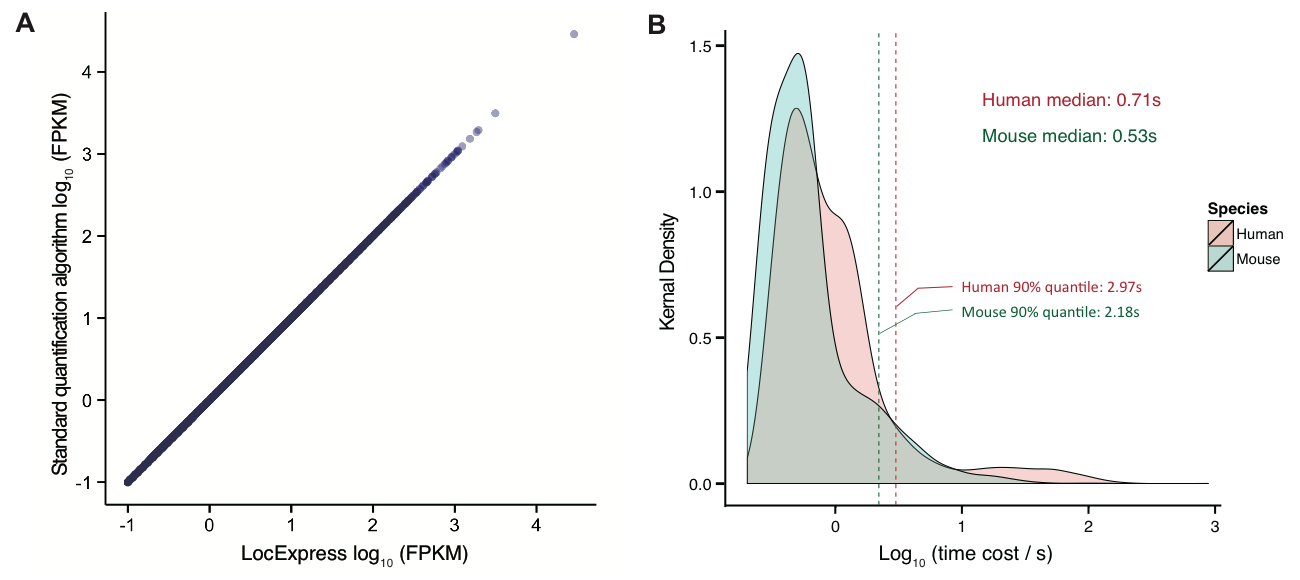


A) LocExpress archives the same accuracy as the standard quantification algorithm.

B) The time cost per sample for a novel transcript. The median time cost is 0.71s in human and 0.53s in mouse. The Linux box is configured with two Intel Xeon Processor E5-2670 v2 10C 2.5GHz CPUs, with 4*16GB ECC DDR3 1866MHz memory.

## Figure S2 – Detailed workflow of LocExpress (Additional file 3)

When a transcript is submitted, LocExpress firstly compares the transcript structure with reference annotations by Cuffcompare (v2.2.1). Then, if the class code is “=” or “c”, it’s considered as a known transcript and the pre-calculated FPKMs are returned directly. Otherwise it’s considered as “novel” and the spanning region is merged with pre-calculated overlapped (with gap no more than 50bp) bundle regions to infer the MSB. In this manner, LocExpress ensures that the MSB is actually the bundle that the transcript belongs to when estimating the expression following the standard pipeline (merge the transcript into reference annotation, and run StringTie through the new merged annotation). Then, reads and gene models in the MSB are extracted to call Stringtie. Finally, the raw FPKMs are corrected and normalized to output to users.

# Additional methods and discussion

## Datasets

LocExpress covers common tissues and cell types in human and mouse. In human, we include 40 samples covering 20 normal tissues / cells, and 28 samples covering 14 cell lines. In mouse, we include 24 normal tissues / cells samples and 9 cell line samples. (see Additional file 2 for the full list.)

## RNA-Seq raw data retrieval

Samples with ID stars with “SRX” or “SRR” were downloaded from SRA. Samples with ID stars with “ENC” were downloaded from ENCODE.

## Reads alignment

We mapped human reads to the genome hg38, and mouse reads to mm10 by HISAT2 (v2.0.2-beta) (Refer to Additional File 2 for the statistics of mapped reads). For six mouse samples with colorspace, Bowtie (v1.1.2) with “-C -y” is used to do the alignment.

## Expression estimation

The expression is estimated by StringTie (v1.2.2) with “-G -e -B -v” based on the reference annotation GENCODE v24 for human and vM9 for mouse. With the option “-v”, we can obtain the bundles of each sample.

## Evaluation on newly added transcripts

We also evaluated the performance of LocExpress on real novel transcripts. To obtain novel transcripts, we first compared the human (v24) and mouse (vM9) GENCODE gene models with these of baseline releases (v21 for human and vM7 for mouse) by Cuffcompare v2.2.1. Then transcripts with class code different from “c” or “=” were considered as “novel transcripts” (3946 in human and 3874 in mouse). These transcripts were removed from the original reference gene models and fed into the LocExpress one by one in all samples, resulting in 2727 human and 3074 mouse transcripts identified as expressed (i.e. FPKMs >= 0.01) in at least one sample. Comparison with the output of StringTie ran in quantification-only mode (specified by “-e -B”) with newly added transcripts removed as reference annotation demonstrated that LocExpress is able to estimate expression abundance correctly for novel transcripts (Figure S1A), in nearly real-time (median time 0.71s for human and 0.53s s for mouse, also see Figure S1B)
