# Supplementary figures and images for: LocExpress: a web server for efficiently estimating expression of novel transcripts
Source: BMC Genomics. 2016 Dec 22;17(Suppl 13):1023. doi: 10.1186/s12864-016-3329-3 (PMC5260097; doi:10.1186/s12864-016-3329-3)

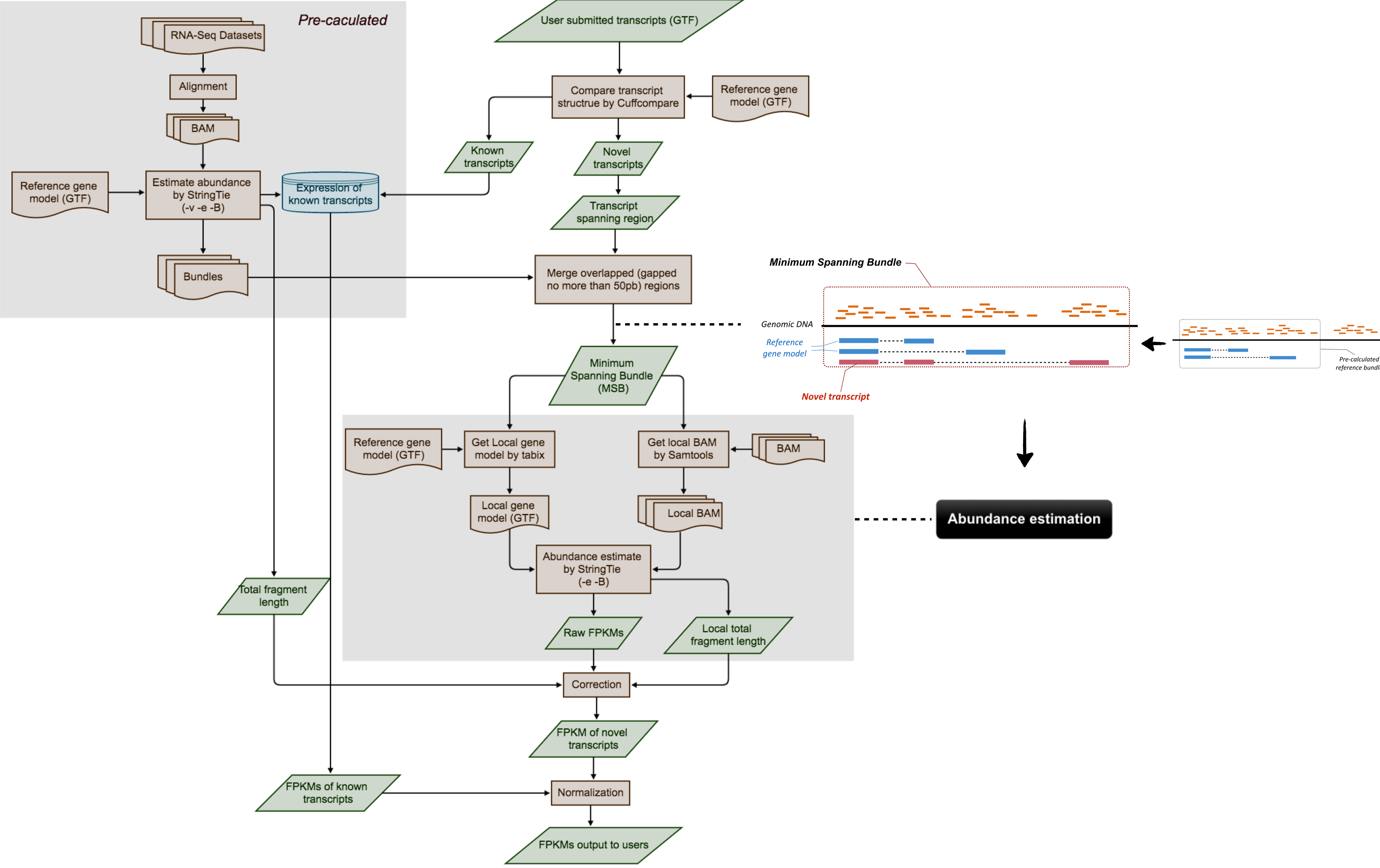

Supplement: Additional file 3: — This file is Figure S2, a flowchart of detailed workflow of LocExpress. (PDF 332 kb) [file 12864_2016_3329_MOESM3_ESM.pdf]
